# Physical fitness of children and youth with asthma in comparison to the reference population: Cross-sectional results of the population-based MoMo study in Germany

**DOI:** 10.1186/s13102-021-00359-0

**Published:** 2021-10-22

**Authors:** Anke Hanssen-Doose, Robert Jaeschke, Claudia Niessner, Doris Oriwol, Annette Worth

**Affiliations:** 1 University of Education Karlsruhe, Bismarckstr. 10, 76133 Karlsruhe, Germany; 2 Rehabilitation Centre for Children With Respiratory Diseases, Fachkliniken Wangen, Am Vogelherd 14, 88239 Wangen, Germany; 3 Karlsruhe Institute of Technology, Engler-Bunte-Ring 15, 76131 Karlsruhe, Germany

**Keywords:** Chronic disease, Health, Cardiorespiratory endurance, Muscular strength, Coordination, Physical activity, Motor performance, Motor abilities, Motor skills, Adolescents

## Abstract

**Background:**

Physical fitness is an essential marker of health. The literature regarding the question of whether individuals with asthma have reduced physical fitness compared to their non-asthmatic peers is inconsistent and focuses on the cardiorespiratory endurance dimension. This study provides a comparison of different dimensions of physical fitness in individuals with and without asthma on the basis of the German population-based study “KiGGS” (German Health Interview and Examination Survey for Children and Adolescents) and its in-depth study “MoMo” (2009–2012: wave 1 and 2014–2017: wave 2).

**Methods:**

In total, 7731 individuals aged 6–30 years were included in this cross-sectional analysis at two measurement waves, including 353 individuals with and 7378 without asthma. The 12-month prevalence of physician-diagnosed asthma was assessed by interview. Physical fitness was measured by six test items of the MoMo test profile. “Cardiorespiratory endurance” was measured by an ergometric test, “muscular strength” by standing long jump, push-ups and sit-ups and “coordination” by jumping sideways and balancing backwards. Because of the broad age range of the sample, age- and sex-specific percentiles were used. Physical activity, age, gender and general state of health were assessed by questionnaire.

**Results:**

The individuals with asthma reported a poorer general state of health at both measurement waves. However, the results of the fitness tests indicated that they were as physically fit as their peers without asthma in relation to cardiorespiratory endurance and muscular strength. The mean percentiles were all within the same range. The results of the comparisons of coordination performance were inconsistent. At wave 1 they were within the same range, at wave 2 individuals with asthma showed a poorer coordination performance (*p* = 0.041; HL = 4.125, CI of HL 0.155–8.125).

**Conclusions:**

To the best of our knowledge, this is the first study to compare the physical fitness of individuals with and without asthma by considering several dimensions of physical fitness. The study demonstrates that cardiorespiratory endurance and muscular strength are not reduced in individuals with asthma. The results of the comparisons at the two measurement waves were remarkably stable.

## Introduction

In childhood and adolescence, physical fitness is an essential marker of health [1–3]. High physical fitness performance in childhood and adolescence is associated with reduced present and future risks of disease and premature death [4, 5]. Most likely, for reasons of limited resources, physical fitness levels are rarely analysed (comprehensively) in health monitoring [6]. To assess the physical fitness levels of younger populations, an appropriate concept must be selected. In this study, a dimension-oriented concept covering a broad age range was selected that consisted of the main dimensions of cardiorespiratory endurance, muscular strength, speed, coordination and flexibility [2, 3].

This paper seeks to examine the physical fitness levels of children, adolescents and young adults with chronic asthma. Asthma is the most common pulmonary disease in children and adolescents and is characterised by recurrent attacks of breathlessness, wheezing and other symptoms [7]. During asthma attacks, the lining of the bronchial tubes swells. This causes less airflow into and out of the lungs [7]. The prevalence of asthma has been globally increasing for decades [7–9], with currently more than 339 million affected individuals [7]. In Germany, the 12-month prevalence of physician-diagnosed asthma in younger populations is stagnant at approximately 4.0% (3.1% in girls versus 5.0% in boys) [10], whereas the lifetime prevalence is documented to be higher at approximately 6.3% (5.2% in girls and 7.4% in boys) [11].

The cause of asthma is thought to be complex. In addition to genetic predispositions, mobility and (changing) environmental exposures that provoke allergic reactions or irritate the airways play a role [7]. The major objective of treatment, therefore, is disease control and reduction of the burden of disease [7, 12]. Asthma is associated with high costs for healthcare systems and society due to school and work absenteeism and therapies [12]. In addition to the burden on society, the disease has a substantial burden on individuals and their families [12]. Sleeplessness during the night and daytime fatigue are typical negative implications [7].

During asthma attacks, physical capacity is reduced in individuals with otherwise normal lung function [9]; apart from this, there are no restrictions on training [13]. Despite the positive effects of regular physical training (less hospitalisation and less medication necessary [13]), individuals with asthma seem to train less frequently and intensively [14]. Family influence or medical advice are discussed as barriers [15]. There are potential explanations for the association of chronic asthma with reduced physical fitness.

A higher state of physical fitness may increase the threshold for developing respiratory symptoms [16], reduce the burden of disease for individuals with asthma and increase their quality of life [15]. Rasmussen et al. [17] found better cardiorespiratory fitness to be associated with better airway reactivity and a reduced risk of developing asthma.

While improvements in cardiopulmonary fitness through physical training are found frequently in individuals with asthma [15], other dimensions of physical fitness, such as muscular strength and coordination, are rarely studied.

Regular physical activity has been found to be a preventative factor for new disease onset of asthma in some studies [18–20]. There is consensus that physical training (as a specific form of physical activity) has proven to be well tolerated in individuals with asthma [13, 15], especially with a medical evaluation before training, adequate monitoring and education [12, 17]. Education comprises, for example, breathing techniques and respiratory exercises.

Studies that directly compare the physical fitness and physical activity levels in children and adolescents with asthma and their non-asthmatic peers have produced inconsistent results. Concerning physical fitness, some studies found children and adolescents with asthma to be less fit than their non-asthmatic peers [21], whereas others reported no difference [22]. With respect to physical activity, some studies reported children and adolescents with asthma to be less active [21, 23], whereas others reported no difference in physical activity [22]. Reduced cardiorespiratory fitness was found by Andrade and colleagues [21] in children with moderate and severe asthma in Brazil using the 6-min walk test. This result was described to be directly influenced by the lack of physical activity of the individuals with asthma. Berntsen et al. [22] came to the opposite findings in Norway and did not find reduced cardiorespiratory fitness or reduced physical activity levels in individuals with asthma in contrast to those without asthma.

To identify potential health gaps, this population-based study focuses on the comparison of the physical fitness of children, adolescents and young adults with asthma and their peers without asthma. In addition to cardiorespiratory endurance, this article focuses on two other components of physical fitness: muscular strength and coordination. To the best of our knowledge, this is the first study to comprehensively compare the physical fitness levels of individuals with and without asthma in the context of relevant influencing factors.

## Methods

### Study population and study design

These cross-sectional analyses are part of the Motorik Modul Study (MoMo), a long-term population-based study on physical fitness and physical activity in Germany that started in 2003 with a baseline measurement and has a cross-sectional and longitudinal approach [24, 25]. The present study focuses on cross-sectional data collected between 2009–2012 (MoMo wave 1) and between 2014–2017 (MoMo wave 2). MoMo is an in-depth study of the long-term KiGGS study (German Health Interview and Examination Survey for Children and Adolescents) conducted by the Robert Koch Institute [26]. The participants of KiGGS were randomly selected from local population registries in 167 sample points throughout Germany using a stratified multistage probability sampling strategy [26]. These sample points were selected according to the structure of federal states and municipalities of the Federal Republic of Germany. Participants in the MoMo study were randomly recruited from the KiGGS sample, which allows the inclusion of data from the KiGGS study in this analysis [27]. Participants were contacted individually and invited to examination rooms near their homes. In these examination rooms, their physical fitness levels and anthropometric measures were tested, and the participants completed questionnaires. For asthma diagnosis, medical data from the KiGGS study were added and analysed.

### Variables

#### Socio-economic status

Socioeconomic status (SES) includes the education, professional status and household income of the participant parents. The different domains were scored on a scale 1–7 and then combined for a score from 3 to 21 [28]. For each wave the sample was then categorised in three groups while using quintiles (low = first quintile | intermediate = second to fourth quintile | high = fifth quintile).

#### Anthropometric measure

Body height was measured with a portable stadiometer (Seca 213, Hamburg, Germany), and body mass was measured with a digital scale (Seca 813, Hamburg, Germany), both without shoes and with light clothing. Based on this, the BMI (kg/m^2^) was calculated. Weight status was defined according to Kromeyer-Hauschild et al. [29].

#### General health status

From the age of 11 years onwards, the general health status was assessed by a one-item scale: “How would you describe your health in general?” The five responses ranged from “very good”, “good”, “fair”, “poor”, to “very poor” [30]. These responses were dichotomised into two categories: “very good/good” and “fair/poor/very poor”. General health status was included in the analysis because of its predictive power for morbidity, mortality and use of health services in adults [31].

#### 12-month prevalence of asthma

Disease-specific data were collected within the KiGGS study by interviews. The questions to determine the 12-month prevalence of asthma were whether a physician had diagnosed asthma and whether it had occurred in the last 12 months. Additionally, information was requested on the use of asthma medication within the last 12 months. The diagnosis of asthma was assumed when physician-diagnosed asthma occurred or when the use of asthma medication was reported within the last 12 months [32].

#### Physical fitness (PF)

Physical fitness was measured by means of the MoMo test profile [24], which is based on the systematisation by Bös [2, 3]. All tests of the profile originate from validated test batteries and were pretested and documented in a published test manual [33]. Within this paper, six test items that covered the three dimensions “cardiorespiratory endurance”, “muscular strength” and “coordination” were chosen; they statistically had the ability to be modelled and calculated as percentiles. “Cardiorespiratory endurance” was measured by an ergometric test assessing the physical working capacity 170 (PWC170, watts at 170 beats/minute by means of the protocol of the World Health Organization [33]. Muscular strength was measured by the standing long jump, push-up and sit-up tasks. “Coordination” was determined by the jumping sideways test (under time pressure) and by the balancing backwards test (under precision pressure). For each task, we calculated age- and gender-specific percentile curves with our own population-based sample using the LMS transformation method according to Cole and Green [34]. A percentile of 1 represents the lowest age- and gender-specific test performance, whereas a percentile of 99 represents the best age- and gender-specific performance [34]. Within this paper, we combine the percentile values within the same dimension and calculate mean values. In this way, a direct comparison between age- and gender-related heterogeneous groups is possible. In the literature, different cut-offs (at or below the 5th, 9th or 25th percentile) have been documented to identify the “at-risk group” [35, 36] with potential daily motor problems or potentially pathological diseases. Physical performance at or below the 15th percentile has been documented for gross motor coordination problems [35]. In this study, the cut-off point was used for all three dimensions of physical fitness and served to compare the odds of being physically unfit in individuals with asthma and their peers without asthma.

#### Physical activity (PA)

To assess self-reported habitual physical activity, the participants completed the MoMo Physical Activity Questionnaire (MoMo-PAQ), which covers different settings and structures of physical activity, such as sports clubs, leisure time, school, daily activities and overall physical activity [37]. Membership in a sports club, which is an essential setting for physical activity in Germany, was also assessed. The questionnaire is reliable (test–retest reliability: ICC = 0.68) [37]. It consists of 28 questions and covers the settings of physical activity in a normal week, without a defined reference period. Because of the broad age range of the study samples of this analysis, the overall physical activity was chosen as adequate outcome parameter referring to the recommendations of the World Health Organization [38]. Overall physical activity was operationalised for days with at least 60 min of physical activity and as a percentage with 60 min of physical activity every day as described by Prochaska, Sallis and Long.

#### Media consumption

Media consumption was determined by interview within the KiGGS core survey by asking about daily amounts of time spent watching TV or films, using a computer and playing console games [39]. For participants up to the age of 10, the parents were asked to estimate the daily amounts, and participants from the age of 11 years and older reported the amounts themselves. The media times were summed and reported in categories. The categories low, intermediate and high media consumption were based on tertiles in relation to the total population of the KiGGS study in the same measurement wave.

### Statistical analysis

The statistical analyses were conducted using IBM SPSS 24 (Chicago, USA). To characterise the study populations with and without asthma, percentages or means and standard deviations (SD) are reported. For the group comparisons, Mann–Whitney U tests and chi^2^ tests were applied to analyse group differences. The unequal sample sizes of the groups did not influence the test results of the Mann–Whitney U test [40]. The required assumption for the chi^2^ of expected frequencies greater than five was met. To describe the effect sizes, the Hodge Lehmann estimator for the median differences and Cramer’s V are reported [40]. Cramer’s V values greater than 0.1 and lower than 0.3 indicate a small effect, values between 0.3 and 0.5 indicate a medium effect, and values greater than 0.5 indicate a large effect [41]. If the 95% confidence interval for the median difference contains zero, an effect cannot be assumed. The group characteristics and PA-related behaviours were stratified by gender. This stratification was not necessary for the outcome variable of physical fitness due to the age- and gender-specific format as percentiles. To evaluate the prevalence of PA-related behaviours and physical fitness, odds ratios were calculated for asthma as the risk factor, and 95% confidence intervals for the odds ratios were reported. Odds ratios are calculated in general to obtain if an assumed risk factor is associated with an outcome variable e.g. a medical condition. In this analysis, the purpose was to identify if asthma is a risk factor for lower physical fitness. If the 95% CI was greater than one, the prevalence was assumed to be associated with the occurrence of asthma.

## Results

In total 7731 individuals aged 6–30 years were included in this cross-sectional analysis at two measurement waves including 353 individuals with asthma and 7378 without asthma. In wave 1 (2009–2012) N = 3471 individuals aged 6–24 years participated in the study: 163 individuals with asthma (4.7%) and 3308 without asthma (95.3%). Accordingly, a total of 4260 individuals aged 6–30 years participated in wave 2 (2014–2017): 190 individuals with (4.5%) and 4070 without asthma (95.5%). In the groups with asthma, 79% (wave 1) and 95% (wave 2) got asthma-specific medication. A description of the characteristics of the study populations is given in Table 1.

**Table 1:** Characteristics of the study populations

Variables	Wave 1 (2009–2012)		Wave 2 (2014–2017)	
	Individuals with asthma (n = 163)	Reference population (n = 3308)	Individuals with asthma (n = 190)	Reference population (n = 4070)
Male (%) | female (%)	59 | 41	49 | 51	53 | 47	49 | 51
Age (mean ± SD)	13.6 ± 4.7	13.3 ± 4.4	14.5 ± 4.9	14.4 ± 4.8
Socioeconomic status (SES*)				
Low (%)	7.2	8.3	7.5	8.5
Intermediate (%)	65.6	65.3	68.1	64.4
High (%)	27.2	26.4	24.4	27.1
BMI (kg/m^2^) (mean ± SD)	19.9 ± 4.2	19.7 ± 4.2	20.9 ± 4.8	20.2 ± 4.2
Weight status**				
Underweight (%)	5.5	6.9	4.9	7.4
Normal weight (%)	80.4	78.8	76.1	76.7
Overweight (%)	7.4	8.2	9.8	10.0
Obese (%)	6.7	6.0	9.2	5.9
General health status***				
Very good/good (%)	84.8	90.6	80.6	91.2
Fair/poor/very poor (%)	15.2	9.4	19.4	8.8

Data are either percent (%) or mean values (mean) ± standard deviation (SD); SES* based on Lampert et al. [28]; weight status** based on Kromeyer-Hauschild et al. [29]; general health status*** based on De Bruin et al. [30]

With respect to age, socioeconomic status, BMI and weight status, the groups did not differ in each wave. Significant differences between the groups were found for gender, with more male individuals in the group with asthma in wave 1 (chi^2^ = 5.573; *p* = 0.018, Cramer’s V = 0.040), and for the general state of health, with poorer general health status in the group with asthma (chi^2^ = 4.063; *p* = 0.044, Cramer’s V = 0.043). Stratified by gender, a difference in the general state of health was found only in males (chi^2^ = 4.056; *p* = 0.044, Cramer’s V = 0.060) but not in females (chi^2^ = 0.771; *p* = 0.380, Cramer’s V = 0.026). After stratification by gender, no other gender-specific differences were detected in wave 1. In wave 2 the difference for the general health status was also significant (chi^2^ = 17.702; *p* < 0.001, Cramer’s V = 0.076) whereas the gender was not to be found different for the group with or without asthma.

Individuals with and without asthma showed similar overall physical activity as measured by at least 60 min of moderate to vigorous physical activity and by the percentage of sufficiently active individuals at both measurement waves. Membership in sports clubs and media consumption also did not differ statistically between the individuals with and without asthma at both measurement waves (Table 2). Stratified by gender, the results were similar.

**Table 2:** Prevalence of PA-related behaviours in individuals with asthma and their non-asthmatic peers

Variables	Wave 1 (2009–2012)		Wave 2 (2014–2017)	
	Individuals with asthma	Reference population	Individuals with asthma	Reference population
Days with at least 60 min PA* (mean ± SD)	3.8 ± 1.8	3.8 ± 1.7	3.7 ± 1.8	3.8 ± 1.7
Sufficiently active** (%)	16.8	13.3	10.9	12.6
Membership sports club (%)	70.4	63.9	60.1	62.6
Low media consumption*** (%)	32.7	36.7	45.1	50.5
Intermediate media consumption (%)	34.0	32.0	32.4	27.1
High media consumption (%)	33.3	31.3	22.5	22.4

Data are mean values (mean) ± standard deviation (SD) or percent (%)

*Days per week with at least 60 min of moderate to vigorous physical activity, **percentage sufficiently active (60 min every day) based on Prochaska et al. [50], media consumption*** based on tertiles in relation to the total population of the KiGGS study in wave 1 and on tertiles in relation to the total population of the MoMo study in wave 2

At Wave 1 (2009–2012), individuals with and without asthma had comparable cardiovascular fitness, muscular strength and coordination (Table 3). The Mann–Whitney U test showed nonrelevant *p* values for all three components of physical fitness, and the Hodge-Lehmann measure of effect size included zero (meaning that no relevant effect was detected). At Wave 2 (2014–2017), individuals with asthma again had comparable cardiovascular fitness and muscular strength, but a slightly lower coordination performance in comparison to the reference population (*p* = 0.041; HL = 4.125, CI of HL 0.155–8.125).

**Table 3:** Physical fitness in individuals with asthma in comparison to their non-asthmatic peers

Variables	Wave 1 (2009–2012)		Wave 2 (2014–2017)	
	Individuals with asthma	Reference population	Individuals with asthma	Reference population
Cardiorespiratory endurance*	48.9 ± 28.4	51.4 ± 28.9	50.0 ± 28.7	53.4 ± 28.0
Mann–Whitney U test	*p* = 0.390; HL = 2.497 (CI − 3.128–8.263)		*p* = 0.155; HL = 3.451 (CI − 1.243–8.349)	
Muscular strength	55.3 ± 24.3	52.6 ± 23.0	47.3 ± 24.9	50.5 ± 23.3
Mann–Whitney U test	*p* = 0.139; HL = − 3.051 (CI − 7.178–0.997)		*p* = 0.060; HL = 3.638 (CI − 0.152–7.434)	
Coordination	52.5 ± 25.5	53.4 ± 24.2	47.6 ± 26.7	51.7 ± 24.3
Mann–Whitney U test	*p* = 0.719; HL = 0.754 (CI − 3.377–4.887)		*p* = 0.041; HL = 4.125 (CI 0.155–8.125)	

*Data are mean percentiles based on Niessner et al. [34] ± standard deviations (SD), *p* values and effect size Hodge Lehmann (HL) with related 95% confidence intervals (CI)

The prevalence of PA-related exposure was not associated with the occurrence of asthma. The odds ratios for being less physically active were not elevated for individuals with asthma (Table 4). Children, adolescents and young adults with asthma did not have an elevated risk of being physically unfit (≤ 15th age- and gender-specific percentile) for their cardiorespiratory endurance and muscular strength (Table 5). The odds of being at risk for coordination problems was not elevated at wave 1, but doubled for the group with asthma at wave 2 (OR 2.013, 95% CI 1.334–3.036).

**Table 4:** Odds ratios (OR) for PA-related behaviours

Prevalence of PA-related behaviours	Wave 1 (2009–2012)	Wave 2 (2014–2017)
	Asthma versus reference population	Asthma versus reference population
Sufficiently active versus not sufficiently active	OR 0.765 (95% CI 0.500–1.170)	OR 1.179 (95% CI 0.734–1.893)
Membership sports club versusnon-membership	OR 0.742 (95% CI 0.500–1.170)	OR 1.113 (95% CI 0.822–1.507)
High versus intermediate or low media consumption	OR 1.099 (95% CI 0.524–1.051)	OR 1.006 (95% CI 0.705–1.436)

Data are odds ratios (ORs) and 95% confidence intervals (CIs)

**Table 5:** Odds ratios (OR) for being physically unfit (≤ 15th age- and gender-specific percentile)

Prevalence physical fitness	Wave 1 (2009–2012)	Wave 2 (2014–2017)
	Asthma versus reference population	Asthma versus reference population
≤ 15th percentile cardiorespiratory Endurance	OR 1.069 (95% CI 0.621–1.838)	OR 1.393 (95% CI 0.876–2.215)
≤ 15th percentile muscular strength	OR 1.214 (95% CI 0.628–2.346)	OR 1.337 (95% CI 0.811–2.205)
≤ 15th percentile coordination	OR 0.891 (95% CI 0.463–1.714)	OR 2.013 (95% CI 1.334–3.036)

Percentiles based on Niessner et al. [34], data are odds ratios (ORs) and 95% confidence intervals (CIs)

## Discussion

The distribution of individuals with asthma at both measurement waves corresponded to the age- and sex-specific general population in Germany, which is attributable to the representative nature of the study. Higher prevalence rates of asthma in males, especially between the ages of 10 and 15 years, have also been found in other population-based studies in Germany [10]. In consideration of the described burden of disease [10], it is not surprising that the group with the diagnosis of asthma reported a poorer general state of health. This “health gap” can be an indicator of worse lung function parameters or participation disturbances. Due to the large-scale character of this large scale study, there were no embedded lung function diagnostics.

Children, adolescents and young adults with asthma in Germany did not have reduced physical fitness levels in the dimensions of cardiorespiratory endurance and muscular strength. Within the dimension coordination, the results were ambiguous depending on the measurement wave (wave 1: no difference, wave 2: small difference).

The finding of similar levels of cardiorespiratory endurance performance in individuals with and without asthma is supported by the findings of Berntsen et al. [22], who measured n = 95 individuals with asthma versus n = 79 without asthma by a treadmill test. This finding is also supported by Santuz et al. [42], who compared n = 80 individuals with asthma with n = 80 non-asthmatic peers using a treadmill test. In contrast, de Andrade et al. [21] found lower levels of cardiorespiratory endurance in n = 40 individuals with asthma (measured as a shorter walking distance within the 6-min walking test) in comparison to predicted values for healthy individuals. The findings concerning muscular strength and coordination performance could not be compared to other studies. As muscular strength [43] and coordination performance (1) are linked to health, this gap in the knowledge should be filled to determine whether health opportunities are (un)equally distributed. It must be noticed that the results in relation to coordination performance were not consistent in the wave 1 and wave 2 measurement of this study while all other outcomes were stable. However, when calculating a more complex model with a multinomial logistic regression with the confounders sex, age, BMI, general health status, physical activity, physical fitness, media consumption and SES this differences can no longer be detected (wave1: Nagelkerkes-R^2^ = 0.085, *p* = 0.292; wave2: Nagelkerkes-R^2^ = 0.032, *p* = 0.092). Therefore, we assume that the differences in coordination performance in wave 2 in the odds ratios either occur due to random effects or due to differences in setting-specific physical activity which we did not cover precisely in the analysis of the influencing factors. Due to the broad age range, we had to choose appropriate physical activity variables with information across all age groups. In this study, overall physical activity, overall organised physical activity and media consumption did not differ between the individuals with and without asthma even though disease-specific obstacles in engaging in physical activity might exist.

Similar levels of overall physical activity in individuals with asthma have also been found in other studies [22, 44, 45]. In a study from the year 2018, n = 65 young adults with asthma reported lower physical activity levels in concerning vigorous physical activity in comparison to controls without asthma. Negative expectations and fear-avoidance beliefs were discussed as relevant predictors of PA levels in young adults with asthma [46]. For future studies analysing the physical fitness levels of children, adolescents and adults with asthma, the influencing factor physical activity should be analysed more differentiated and the amount and proportion of vigorous physical activity should be included into the analysis.

In all groups of this study, only a small percentage met the 60-min threshold of moderate to vigorous physical activity every day (wave 1: 16.8% asthma, 13.3% non-asthmatic reference group; wave 2: 10.9% asthma, 12.6% non-asthmatic reference group). This finding is in line with many other studies; Tremblay et al. [47] refer to it as a “physical inactivity crisis”. Insufficient movement neglects to compensate for the health gap, especially in individuals with chronic diseases.

A possible explanation for the non-existent differences in physical fitness levels in most dimensions between individuals with and without asthma is adequate pharmacotherapy (a large proportion of the individuals with asthma receive asthma-specific medication) and education, which might have had a compensating effect. Education was not assessed in this population-based study, therefore this hypothesis cannot be proved. But it is known from the literature that one-third of all children and adolescents with asthma in Germany received special education between 2010 and 2017. They joined the structured and evaluated patient education programme offered by a patient education network all over Germany [48]. The main topics of the programme are knowledge about asthma, symptoms, self-awareness, avoidance of asthma triggers, breath control, inhalation techniques, management of acute dyspnoea and knowledge about physical training [49]. The continuation of quality-assured education programmes seems promising and offers new tailored education programmes for teachers and trainers. Doctors, clinicians, public health actors, teachers and trainers should cooperate (more) closely to support children, adolescents and young adults with asthma to become and remain physically active and fit.

There are several limitations of the study that must be considered. The two groups were formed by the variable of having or not having physician-diagnosed asthma, assessed by interview. This is a weak diagnostic assessment, but due to the population-based character and limited resources, it is an unavoidable limitation. Presumably, individuals with severe forms of asthma and poor disease control did not participate equally in the KiGGS-/MoMo study in comparison to those with mild forms and healthy individuals. At the same time, in the group without asthma, there is likely a proportion of individuals with undiagnosed asthma. For future smaller comparisons, a parameter of lung function should be included. The cut-off point at or below the 15th percentile needs to be questioned in future studies to find the most accurate threshold. We chose this cut-off because of the position between the 5th and 25th percentiles, as elaborated in former studies. The assessment of physical activity realised by questionnaire could be associated with a lack of precision. The major strengths of the present analysis are the large sample sizes, the population-based character of the study and the objective and comprehensive comparisons of physical fitness, including the components of cardiorespiratory endurance, coordination and muscular strength. Furthermore, the analogue measurement of outcome parameters and influencing factors in both groups is worth to be mentioned.

## Conclusions

The present population-based study provides a comparison of the physical fitness levels of individuals with and without asthma, focusing on cardiorespiratory endurance, muscular strength and coordination performance as well as physical activity levels. To the best of our knowledge, no other studies have compared individuals with and without asthma within these three health-related dimensions of physical fitness in the context of relevant influencing factors. The results of the comparisons at the two measurement waves of the population-based study were remarkably stable except for the coordination performance. In this study, individuals with asthma were as physically fit in relation to cardiorespiratory endurance and muscular strength and they were as physically active as their peers without asthma. Future studies should deepen the evidence in relation to coordination performance and should explore how pharmacotherapy and education influence physical capacity and physical fitness. It can be perceived negatively that only 16.8% (wave 1) and 10.9% (wave 2) of the individuals with asthma met the 60-min threshold of moderate to vigorous physical activity every day. This indicates lost potential to positively influence health and health-related quality of life in order to overcome the health gap.

## Data Availability

The datasets generated and/or analysed during the current study are not publicly available because the Federal Office for the Protection of Data explicitly forbid making the data publicly available because informed consent from the study participants did not cover the public deposition of data. The datasets are available from the corresponding author on reasonable request.

## Abbreviations

KiGGS: German health interview and examination survey for children and adolescents
MoMo: Motorik Modul
HL: Hodge Lehmann (effect size)
SD: Standard deviation
SES: Socioeconomic status
BMI: Body mass index (kg/m^2^)
PWC 170: Physical working capacity 170
MoMo-PAQ: MoMo physical activity questionnaire
CI: Confidence interval
PA: Physical activity
OR: Odds ratios
